# STAGES AND FACTORS OF THE “PERIOPERATIVE PROCESS”: POINTS IN COMMON WITH THE AERONAUTICAL INDUSTRY

**DOI:** 10.1590/0102-672020180001e1423

**Published:** 2019-02-07

**Authors:** Carlos Federico DAVRIEUX, Mariano PALERMO, Edgardo SERRA, Eduardo Javier HOUGHTON, Pablo Agustín ACQUAFRESCA, Caetano FINGER, Mariano Eduardo GIMÉNEZ

**Affiliations:** 1 Fundación DAICIM (Docencia, Asistencia e Investigación en Cirugía Invasiva Mínima, Buenos Aires, Argentina

**Keywords:** Surgical procedur, Perioperative perio, Aviatio, Safety, Procedimento cirúrgic, Período perioperatóri, Aviaçã, Segurança

## Abstract

**Background:**

The aeronautical industry is one of the disciplines that most use control systems. Its purpose is to avoid accidents and return safer flights. The flight of an airplane, from its takeoff to its landing is a process divided into stages under strict control. A surgical procedure has the same characteristics. We try to identify and develop the stages of the surgical process using the experience of the aviation industry in order to optimize the results and reduce surgical complications.

**Aim:**

To identify and develop the stages of the surgical process so that they could be applied to surgery departments.

**Methods:**

A search, review and bibliographic analysis of the application of aeronautical control and safety to medical practice in general and to surgery, in particular, were carried out.

**Results:**

Surgical process comprises the perioperative period. It is composed of Preoperative Stage (it is divided into 2 “sub-steps”: hospital admission and control of preoperative studies) Operative Stage (it is divided into 3 “sub-steps”: anesthetic induction, surgery, and anesthetic recovery) and Postoperative Stage (it is divided into 2 “sub-steps”: control during hospitalization and ambulatory control). Two checkpoints must be developed. Checkpoint #1 would be located between the preoperative and operative stages, and checkpoint #2 would be located between the operative and postoperative stages. Surgical factors are surgeons, instrumental and technology, anesthesiology and operating room environment.

**Conclusion:**

It is possible and necessary to develop a systematic surgical procedure. Its application in the department of surgery could optimize the results and reduce the complications and errors related to daily practice.

## RESUMO

**Racional:**

A indústria aeronáutica é uma das disciplinas que mais utiliza sistemas de controle. Sua finalidade é evitar acidentes e retornar voos mais seguros. O voo de um avião, desde a decolagem até a aterrissagem, é processo dividido em etapas com estrito controle. Um procedimento cirúrgico tem as mesmas características. Tentar identificar e desenvolver etapas no processo cirúrgico, utilizando a experiência da indústria aeronáutica, poderá otimizar os resultados e reduzir as complicações cirúrgicas.

**Objetivo:**

Identificar e desenvolver etapas no processo cirúrgico para que possam ser aplicadas nos serviços de cirurgia.

**Métodos:**

Foram realizadas pesquisas, revisão e análise bibliográfica sobre o controle e segurança aeronáutica e aplicando-as na prática médica em geral e à cirurgia em particular.

**Resultados:**

O processo cirúrgico compreende o período perioperatório. É composto de pré-operatório (dividido em duas sub-etapas: admissão hospitalar e controle de estudos pré-operatórios); fase operatória (dividida em três sub-etapas: indução anestésica, operação e recuperação anestésica) e fase pós-operatória (dividida em duas “sub-etapas”: controle durante a hospitalização e controle ambulatorial). Dois pontos de verificação devem ser desenvolvidos. O ponto de checagem nº 1 estaria localizado entre os estágios pré-operatório e operatório, e o ponto de checagem nº 2 entre os estágios operatório e pós-operatório. Fatores cirúrgicos são cirurgiões, instrumental e tecnologia, anestesiologia e ambiente de sala de cirurgia.

**Conclusão:**

É possível e necessário desenvolver um procedimento cirúrgico sistemático. Sua aplicação no departamento de cirurgia poderia otimizar os resultados e reduzir as complicações e erros relacionados à prática diária.

## INTRODUCTION

The purpose of the surgical act is to improve the health of patients. The perioperative period is the time lapse surrounding the surgical act. It is subdivided into three stages: preoperative, operative and postoperative. They must fulfill specific actions to achieve their final objective. It is a “process”^6^.

At present several disciplines apply systems to control their activities. It aims to achieve the expected results successfully. The aeronautical industry is one of the disciplines that most use these control systems. It gives a fundamental role to both human resources (pilot training, simulation, training, etc.) and aircraft (constant revision and repair of engines and aerodynamics, updating of autopilot programs, application of technologies, etc.), as well as to routine procedures (control of passengers and their luggage at the airport, checklist in the pilot cabin, flight route, etc.). Its purpose is to avoid accidents and return safer flights. Every accident is a chain of unfortunate events. Isolated, no event is fatal. Its implementation reduced drastically the incidence of air crash accidents^5^.

In surgery, an accident results in perioperative complications and/or poor results. In addition, it increases costs considerably^7^. For each surgical procedure, there is a certain number and type of complications, associated with human errors, defects in the instruments used, or failures in routine processes (for example mistakes during hospitalization that can trigger an error when designating the surgical side of the patient operated on inguinal hernia). It is a process formed by several stages. Completing one of them suboptimal or unsatisfactorily compromises the result of the next. In case of applying checkpoints, these problems would be detected. If at that time the course of the process cannot be corrected, it continues with an “error carry” system. The end would be a complication or a poor surgical result.

In this manuscript, we try to identify and develop the stages of the surgical process using the experience of the aviation industry to be easily applied to the surgery departments in order to optimize the results and reduce surgical complications.

The objective of this manuscript is to identify and develop the stages of the surgical process so that they can be applied to surgery departments.

## METHODS

A search, review and bibliographic analysis of the application of aeronautical control and safety to medical practice in general and to surgery, in particular, were carried out. Medline, Embase, and SciELO were used as search sites. The headings searched were “aviation safety”, “aeronautic safety” and “surgical safety”. The use of boolean operators optimized the survey. There were no restrictions regarding the date and type of published studies. Were analyzed the articles considered to be influential for the improvement of surgical safety.

## RESULTS

Twenty-one articles were analyzed, being 12 reviews and nine clinical studies. Ten were selected because they showed association between the aeronautical industry and the surgery. Many articles were addressed to the application and result of the surgical safety checklist. Some studies analyzed the importance on the training process that operators must have in order to carry out the correct practice. In other cases, we studied how routine processes were optimized by using systematic controls in stages.

**FIGURE 1:**
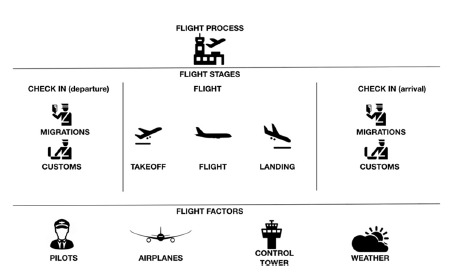
Graph showing the “flight process”: it allows to observe the characteristics of the procedure developed in an airplane flight, and its influential factors

The analysis of the reviewed literature have allowed the development of a system of controls by stages applicable to the surgical process, imitating aeronautical safety. A flight, like a surgery, is a long and complex process carried out by one “teamwork”^20^. Its control is essential to avoid accidents. The aeronautical industry has understood this perfectly, and its philosophy can be transferred to the surgical field. In this way, it can be understood that the “flight process” is divided into stages (Figure 1)^14^.

The first of these could be called “departure check-in”, the second “flight” and the third “arrival check-in”. In the same way, the “surgical process” is adapted, which can be divided into a first “preoperative” stage, a second “operative”, and a third “postoperative”. In turn, each of them is subdivided into “sub-stages”, also intrinsically related. At the end of the stages, the controls must be applied to determine if the objectives were achieved satisfactorily. Then, move on to the next stage. Each stage has characteristics that depend on factors (human resources, technology, environment) intervening at that precise moment. The intervening factors in aeronautics (pilot, airplanes, control tower, weather) can also be compared with surgery (surgeon, instrumental, anesthesiologist, operation room environment)^9^.

### Analysis of the “surgical process”

**FIGURE 2:**
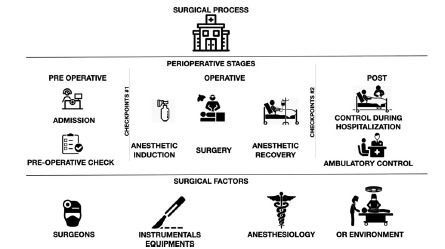
Graph showing the characteristics of the “surgical process” and the “surgical factors”

It comprises the total of the perioperative period. Its ultimate goal is the improvement of the patient’s health through surgery. It is composed of three “stages” (Figure 2).

### A) Preoperative Stage 

It is the first stage of the perioperative period. Its objective is the preparation of the patient for surgery. It is divided into 2 “sub-steps”.

#### Sub-step #1: Hospital admission

It should be carried out by trained administrative personnel. Its objectives are the administrative admission of the patient, their correct identification, and their hospitalization in the corresponding unit (general hospitalization room, Coronary Unit, Intensive Care Unit, others)^12^.

#### Sub-step #2: Control of preoperative studies

It should be carried out by surgery residents or plant surgeons, with knowledge of the surgery to which the patient will be submitted. Its objectives are to control the blood tests required (blood count, hepatogram, ionogram, renal function, coagulogram, others), presurgical cardiology studies (electrocardiography, others), imaging studies (radiography, ultrasound, tomography, resonances, others), material applied prosthetic (prosthetic meshes for hernioplasties, mechanical sutures, other devices), among others (spirometry, endoscopy, etc.).

### B) Operative Stage 

It is the second stage of the perioperative period, composed of three “sub-steps”. The goal is to successfully carry out the patient’s surgery. It should be noted that, once the patient is in the operating room (OR), the protagonists of the surgical act should maintain the “operating room asepsis”. This not only includes avoiding bacterial contamination of the surgical environment but also keeping conversations that are straightly related only to the operative procedure. This concept, used in the aeronautical industry and applied moments before starting a flight, is called “cockpit asepsis”.

#### Sub-step #1: Anesthetic induction

It should be carried out by an anesthesiologist and an anesthesia technician, together with an assistant, if necessary. The objective is to perform the anesthesia required by the patient, according to the procedure to be performed (local, regional or general anesthesia). It would be a good practice for the surgeon to be present at this moment, not only to transmit tranquility to the patient, but also to offer help to the anesthesiologist in case of difficult intubation and to be aware of the drugs administered for anesthesia.

#### Sub-step #2: Surgery

The objective of any surgical procedure is to solve the health problem that afflicts the patient. For this reason, the tactic must be clear (for example laparoscopic cholecystectomy with intraoperative cholangiography) and also the operative technique (for example French position). The tactic should be defined prior to the time of surgery (for example patient with acute lithiasis cholecystitis of four days of evolution, early or late cholecystectomy?). The technique is inherent to the operator and is given by experience and training.

#### Sub-step #3: Anesthetic recovery

It should be carried out by an anesthesiologist and an anesthesia technician, together with an assistant, if necessary. The objective is to perform the anesthetic recovery of the patient. It would be a good practice if the surgeon could be present at this time to help the anesthesiologist in case he needs it. It also allows contact with the patient as soon as he regains consciousness.

### C) Postoperative Stage 

It is the third stage of the perioperative period. The goal is to successfully complete the surgery. It is composed of 2 “sub-steps”.

#### Sub-step #1: Control during hospitalization

The objective is to detect, early, any sign or symptom of an immediate postoperative complication. The wound controls, the cures, the medication administered (analgesics, antiemetics, gastric protectors, antibiotics, antithrombotic prophylaxis), diet and rest should be strict.

#### Sub-step #2: Ambulatory control

At the time of hospital discharge should provide the necessary advice for the welfare of the patient (wound controls, healing, oral medication, diet and rest), as well as schedule the day, place and time of the next consultation. The goal is to avoid and detect any mediate postoperative complication.

### “Checkpoints”

Two checkpoints must be developed, located between stages (Figure 2). Their objective would be to determine if they meet the necessary requirements. It would be possible to know under what conditions the patient advances to the next stage and detect problems to be corrected early.

#### Checkpoint #1

It would be located between the preoperative and operative stages. It must be done by plant surgeons or residents who know the surgical procedure that will be performed. The time of the request would be after the hospitalization. Its objective would be to control that the preoperative stage has been completed correctly (patient identification, preoperative studies, corresponding surgical procedure, surgery´s side, prosthesis). It would avoid and correct errors before the operative stage.

#### Checkpoint #2

It would be located between the operative and postoperative stages. It must be done by plant surgeons or residents who know the surgical procedure performed. The time of the request would be after leaving the OR. Its objective would be to control if operative stage has been completed correctly (patient identification, correct surgical procedure, specific care, medical indications). It would avoid and correct errors before the postoperative stage.

### Analysis of “surgical factors”

#### Surgeons

They should obtain a certification from the society that brings together the surgeons of the region, endorsed by a state entity. In addition to completing the corresponding residence, it is increasingly necessary to complement the development of skills in specialized training centers^18^ ^,8^. It is probable that in the future these practices in simulators will be obligatory, pilots practice in flight simulators. They train both in the aircraft they use daily and in new aircrafts. They test their skills at different flight stages, and are exposed to frequent and infrequent emergencies. In this way, they develop more and better skills. Surgeons will be certified, in the same way as the flight hours of professional pilots^17^. Before the surgery, complex cases should be discussed in a multidisciplinary Athenaeum. Within OR, during surgery, some complications may appear. The surgeon is the team leader. It is the professional who must make the final decision in case of doubt. In the same way, as in the aircraft cockpit, the captain makes decisions regarding the flight.

#### Instrumental and technology

The surgical evolution of the last years inclined toward the so-called minimally invasive surgery. This field encompasses laparoscopic, endoscopic, robotic and image-guided surgery. It is vital to have the instruments in good condition and the correct technology to carry out the procedures successfully. The correct use of disposable or reusable devices is fundamental, and their evaluation of cost-effectiveness according to the characteristics of the institution^15^. The control and periodic review of the systems used (for example: the quality of the image provided by the monitor during a laparoscopy, the pressure and flow offered by the pneumoperitoneum system, or the power of the electrocautery, among other details), are as important as the surgeon’s skill in carrying out the operation. Aircraft are checked before taking off and after landing, on each flight, to determine that all systems are functioning correctly. Anaesthesiology teams, laparoscopy columns and surgical energy-based devices should meet the same requirement.

#### Anesthesiology

The control tower orders air traffic indicating when an aircraft can take off and land. It also offers information about the weather to pilots^5^. The department of anesthesiology that acts during the operation is the control tower of the surgeon, informing him about the vital parameters of the patient (heart rate, respiratory rate, temperature, blood pressure, diuresis), as well as the laboratory data (blood count, acid state-base, coagulogram) and blood loss, among others. Any alarm signal detected by the team of anesthesiologists must be transmitted to the operating surgeon so that he/she can make the pertinent decisions according to the case. This can happen at any time during the perioperative process.

#### Operating room (OR) environment.

Meteorological weather (cloudiness, wind, humidity, temperature, atmospheric pressure) is one of the most important variables that affect aviation since it can condition the takeoff or landing of an aircraft, as well as force a flight route to be modified. In surgery, this factor can be compared to the work environment within an operating room. Although the ideal would be for the actors in this field to work together for a long time so that they know their customs and trust each other, it is not always possible to comply with this requirement. Problems between colleagues and co-workers can generate a hostile work environment, negatively interfering with the outcome of the surgery. It is in this field where non-technical skills (communication, teamwork, decisions, conflict management) and emotional intelligence (EI) must be put into play^16^ ^,^ ^10^.

These skills must be trained and acquired by team leaders.

## DISCUSSION

The flight of an aircraft is a complex process. It is divided into stages allowing be controlled more efficiently. The most influential factors in a flight are pilots, airplanes, weather and communications (control tower). These processes and factors can be extrapolated and compared with surgery.

A surgical procedure is a complex process that involves several people and stages. It aims to improve the health of patients. This complexity means that it must be analyzed by parts, differentiating stages closely related to each other in a correlative way. Each “stage” must meet basic standards so that the desired objectives can be correctly achieved and the next step made in the best possible way. The final results of the succession of these stages should be the satisfactory fulfillment of the surgical procedure understood as “process”.

Many authors consider that the system of procedures applied in the aeronautical industry has advantages that may be useful for surgical practice. Among the most influential, standardized procedures can be enumerated with the application of a control system, the training and constant training of surgeons, and the incentive to teamwork with collective successes^13^. However, others consider that the aeronautical safety control system is not applicable to surgery, especially in cases of emergency^3^.

One investigators group is involved in every aviation accident. This commission includes personnel from the manufacturer of the aircraft, engines, the airline company and an independent government agency (National Transportation Safety Board [NTSB])^11^. Conclusions regarding the accident are formed by all interested parties. It allows finding responsibilities and issuing recommendations to make flights safer.

Many studies report that the use of the surgical safety checklist reduces both morbidity and operative mortality, as well as intraoperative delays^1, 2^. Despite these, its use is still controversial^19^. However, it cannot be differentiated if the improvement in surgical results is due exclusively to the use of the checklist or to the hospital context in which it was applied. Currently, surgical safety questionnaires can be used, especially in minimally invasive procedures^4^. This type of tools should be interpreted only as one more link in the patient safety system.

Just as there is a relationship between the characteristics of an airport and its safety^21^, there could be a relationship between the characteristics of a hospital (location, budget, patient flow) and the safety of patients’ health.

It is currently accepted that the implementation of technology improves aeronautical safety. In surgery, the concept is usually the same. However, the use of new technological devices that are accompanied by a new operative technique requires a learning curve which must be completed correctly to maintain safety standards. In much of the world, the surgeon’s training is given by the residence program. This offers theoretical and practical contents in a period of 3-5 years. But not in all hospitals where surgery residences are developed, the contents are comprehensively addressed. Some have deficiencies, which must be replaced by theoretical-practical courses in simulators. On the other hand, the continuous advance of medicine requires constant updating. That is why the acquisition of skills in surgery is something fundamental in the current professional.

## CONCLUSION

The aeronautical industry uses controls that allow flights to be increasingly safe. It is possible and necessary to develop a systematic surgical procedure, which should be understood as a process composed of well-defined and interrelated stages, whose previous stage result can influence the outcome of the next stage, and could finally determine the success of the surgery in question. The application of a systematic surgical procedure of this type in the department of surgery could optimize the results and reduce the complications and errors related to daily practice.
